# Mechanical characteristics of mesenchymal stem cells under impact of silica-based nanoparticles

**DOI:** 10.1186/1556-276X-9-284

**Published:** 2014-06-05

**Authors:** Irina V Ogneva, Sergey V Buravkov, Alexander N Shubenkov, Ludmila B Buravkova

**Affiliations:** 1 Department of Molecular and Cell Biomedicine, State Scientific Center of Russian Federation Institute of Biomedical Problems of the Russian Academy of Sciences, Khoroshevskoyoe shosse, 76a, Moscow 123007, Russia; 2 I.M. Sechenov First Moscow State Medical University, Moscow 119991, Russia; 3 Faculty of Fundamental Medicine, Lomonosov Moscow State University, Moscow 119192, Russia

**Keywords:** Cell stiffness, Actin cytoskeleton, Cytotoxicity

## Abstract

Silica-based nanoparticles (NPs) pose great potential for medical and biological applications; however, their interactions with living cells have not been investigated in full. The objective of this study was to analyze the mechanical characteristics of mesenchymal stem cells when cultured in the presence of silica (Si) and silica-boron (SiB) nanoparticles. Cell stiffness was measured using atomic force microscopy; F-actin structure was evaluated using TRITC-phalloidin by confocal microscopy. The obtained data suggested that the cell stiffness increased within the following line: ‘Control’ - ‘Si’ - ‘SiB’ (either after 1-h cultivation or 24-h incubation). Moreover, the cell stiffness was found to be higher after 1-h cultivation as compared to 24-h cultivation. This result shows that there is a two-phase process of particle diffusion into cells and that the particles interact directly with the membrane and, further, with the submembranous cytoskeleton. Conversely, the intensity of phalloidin fluorescence dropped within the same line: Control - Si - SiB. It could be suggested that the effects of silica-based particles may result in structural reorganization of cortical cytoskeleton with subsequent stiffness increase and concomitant F-actin content decrease (for example, in recruitment of additional actin-binding proteins within membrane and regrouping of actin filaments).

## Background

Nanoparticles (NPs), based on pure crystalline silica (Si), are capable of fluorescence detection, which makes them applicable as a biological probe [1]. Their high biocompatibility allows these particles to be considered as candidates for providing direct drug delivery [2]. The boron-doped silica NPs are of special interest, as they can be used for boron neutron capture therapy in the treatment of a number of oncological diseases.

However, interactions between NPs and cells (particularly with progenitor cells) have not been elucidated yet. Pi et al. [3] investigated the impact of selenium NPs on the biomechanical properties and F-actin structure of MCF-7 cells, using atomic force microscopy (AFM) and confocal microscopy. The results indicated that adhesion force and Young's modulus, as well as F-actin fluorescence, significantly decreased after these cells had been cultured in the presence of selenium NPs (at concentrations of 2.5 and 5 μg/mL) for 24 h. Similar results were obtained by Xu et al. [4] in cultures of rat cortical neurons in their interaction with silver NPs. F-actin, as well as a β-tubulin fluorescence decrease, was found to be statistically significant and dose-dependent (within a NP concentration range of 1 to 10 μg/mL). Gupta et al. [5] evaluated human fibroblast cell culture treated with gelatin NPs. It was shown that NPs with a size of 50 nm easily diffused through the cell membrane but did not exert their cytotoxic action (it was supported by high cell survival rates and normal ultrastructure at a concentration up to 500 μg/mL). However, when NPs were phagocytosed, vacuoles appeared which, according to the authors' opinion, might destroy structures of the cell cytoskeleton [5]. Allouni et al. [6] demonstrated that TiO_2_ nanoparticles penetrated into L929 fibroblasts either under exposure or even in the absence of the relevant concentrations of cytochalasin D. According to the data obtained by L'Azou et al. [7] in a culture of renal epithelial cells, cytotoxicity of TiO_2_ NPs is strictly dose-dependent and can be explained by the initiation of oxidative stress in cells.

Thus, issues concerning NPs' interactions with membrane and the submembranous cytoskeleton have not been profoundly clarified. The membrane is the main cell structure, which mediates the primary interactions between the cell and the environment. Changes in membranous structure as well as alterations of the cortical cytoskeleton (which is inseparably linked to phospholipid bilayer) may launch a number of intracellular processes, while changes in the cortical cytoskeleton may initiate a number of signaling pathways and regulate the activity of ion channels. By means of patch clamp techniques, it was shown that actin microfilaments, which formed the structure of the cortical cytoskeleton, participated in the regulation of chloride ion channels [8,9], Na^+^/K^+^-ATPase [10], voltage-gated sodium channels in brain cells [11], and sodium channels in the cells of polar reabsorption epithelium [12]. Disintegration of actin filaments with cytochalasin D resulted in activation of sodium channels in the K562 cell line; actin polymerization on the cytoplasmic side of the outer cell membrane induced their inactivation [13]. Moreover, fragmentation of actin filaments (associated with the plasmatic membrane), after being induced by cytosol actin-binding Ca^2+^-sensitive protein (similar to endogenous gelsolin), may constitute the main factor, enhancing the activity of sodium channels in response to an increase in intracellular calcium ion concentrations in the K562 cell line [14,15]. Furthermore, actin can be transferred from the membranous to the cytoplasmic fraction in the form of F-actin with further dissociation of the latter to G-actin, as well as directly in the form of G-actin. A transient increase in G-actin content, in turn, may initiate some signaling pathways (for instance, some serum response factor (SRF)-dependent pathways) [16].

However, evaluation of changes in the structure of the membrane and the submembranous cytoskeleton is strongly altered by their fixing or can be very time-consuming, which may result in some uncharacteristic changes of the cytoskeleton. At the same time, mechanical characteristics of cells (particularly their stiffness) can be used as the measure of their intact structure. Measurements of the mechanical characteristics of cells can be performed *in vivo* within a short period of time using AFM.

In view of the above, the main objective of this study was to determine the mechanical characteristics of mesenchymal stem cells when cultured in the presence of silica and silica-boron nanoparticles.

## Methods

### Isolation of mesenchymal stem cells and their cultivation conditions

In order to obtain the primary culture, a method of enzymatic processing of the stromal vascular fraction isolation from human lipoaspirates was used [17,18]. The obtained cells were cultivated in α-MEM medium (MP Biomedicals, Santa Ana, CA, USA) with 2 mM of glutamine (PanEco, Moscow, Russia), 100 IU/mL of penicillin, 100 μ/mL of streptomycin (PanEco), and 10% fetal bovine serum (Hyclone, Logan, UT, USA) added to the culture. The cell seeding density was 3 × 10^3^ cells/cm^2^. Standard cultivation was performed at 37°C and under 5% CO_2_ using a CO_2_ cultivator (Sanyo, Moriguchi, Osaka, Japan). The cells of passages 3 to 5 were used for the experiments. Silica (Si) and silica-boron (SiB) NPs were added to the culture medium at the same concentration of 100 μg/mL. Cultivations were performed for 1 and 24 h. Nanoparticles were prepared at the Prokhorov General Physics Institute RAS by the method described in detail previously [19].

### Evaluation of mesenchymal stem cell viability

The proportion of AnV + cells (early apoptosis), AnV+/PI + cells (post-apoptotic necrosis), and PI + cells (necrosis) was determined using an Annexin V-FITC/PI kit (Beckman Coulter, Brea, CA, USA) and Epic XL flow cytofluorimeter (Beckman Coulter) in strict accordance with the standard procedure stated in the manufacturer's manual. At least 10,000 events were analyzed.

### Atomic force microscopy

Atomic force microscopy (AFM) is a useful tool for studying cell mechanics [20,21]. Measurements of transversal stiffness in this study were conducted using a Solver P47-Pro instrument (NT-MDT, Moscow, Russia), in accordance with a technique which has previously been described in detail [22]. For each cantilever, the stiffness (N/m) was adjusted using the resonance position. When working in liquid, soft cantilevers were used with the stiffness coefficient of approximately 0.01 N/m. The contact mode was applied to record the force curves. The radius of curvature (*r*_c_) of the tips of all cantilevers used was assumed to be of 10 nm.

Mechanical characteristics of cells were determined by obtaining the calibration force curve on the glass first in order to calculate the coefficient, which converts cantilever deflection expressed in units of current into units of distance-*a* (m/A). Then, the force curves were recorded on cells, obtaining the ratio *y*(*x*), where *y* is the measured cantilever deviation (A) and *x* is the generalized indentation depth (m). Further, the actual indentation depth and the force applied to it were calculated using the following formulae: *h*_*s*_ = *x* - *y* · *a*, *F*_*x*_ = *y* · *a* · *k*_c_, where *h*_c_ is the actual indentation depth (m), *F*_*x*_ is the actual force applied to a cell (N), and *k*_c_ is the cantilever stiffness coefficient. Finally, at the indentation depth of 60 nm, the change of applied force was determined and the stiffness of a sample was estimated using the following formula: *k*_s_ = *F*_*x*_/*h*_s_.

The obtained results were processed using MATLAB 6.5 software, which was specially developed for this research.

### Confocal microscopy

Structures of fibrillar actin (F-actin) were detected using standard TRITC-phalloidin (Sigma, St. Louis, MO, USA) staining. Cells that had previously been washed off the medium were fixed with 4% paraformaldehyde solution for 15 min. In order to permeabilize the cells, 0.1% Triton X-100 (Sigma) detergent was added to the prefixed cells for 15 min. Then, the cells were rinsed twice with phosphate-buffered saline (PBS). Further, TRITC-phalloidin was added to the cells at a concentration of 50 μg/mL and cultured at 37°C for 40 min. Then, the cells were rinsed thrice with PBS. In order to maintain the fluorescence, the samples were covered by the specific water-soluble Fluoroshield medium containing DAPI (Sigma) to achieve fluorescent staining of DNA. Changes in the structure of actin microfilaments were evaluated using the method of fluorescent microscopy and by using an LSM 780 (Carl Zeiss, Oberkochen, Germany) confocal microscope.

**Figure 1:**
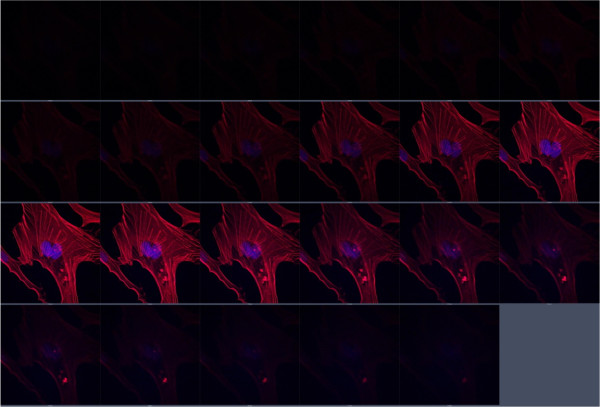
**Typical series of focal optical sections along the vertical axis (control MSCs in this very case).** Red stain appeared due to TRITC-phalloidin and blue stain due to DAPI. Section increment is 0.34 μm.

A coherent laser to produce fluorescence of the DAPI- and TRITC-phalloidin-stained cells (at a wavelength of 355 nm) and an argon laser (at a wavelength of 488 nm) with a power output of 2% (0.5 mW; barrier filter, 355 nm for DAPI and 458/561 nm for TRITC) were used. Registration was performed within blue (401 to 556 nm) and red (566 to 692 nm) spectral regions, using a Plan-Apochromat 63×/1.40 Oil DIC M27 objective. All images were obtained under the same conditions of excitation and registration (laser energy output, detectors' sensitivity, scanning time, etc.) for further densitometric analysis. The average intensity was evaluated within the red channel in each image after performing the background removal. As a result, the average intensity of the red channel was estimated inside each cell. Quantitative analysis of fluorescence intensities was carried out after performing the background removal in each image using the image processing Sigma Scan Pro 5.0 (SPSS, Chicago, IL, USA) software.Assessment of actin fiber distribution within the thickness of a cell was performed using z-stacking (serial focal optical sections along the vertical axis) (Figure 1). Distribution of TRITC-phalloidin fluorescence intensity was measured within each section. In order to avoid any dependence of the obtained result from the selection of initial and final vertical points of a cell scanning, the obtained curves of distribution of fluorescence intensity were normalized according to their maximum values (modes).

Statistical processing of the results was performed using Excel 2007 software for Windows.

## Results

### Evaluation of mesenchymal stem cell viability

**Figure 2:**
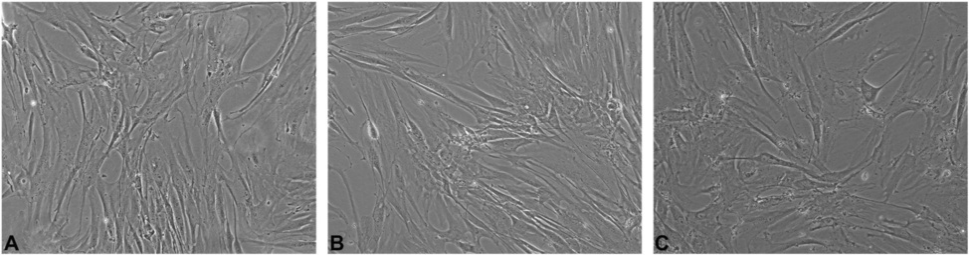
**Typical appearance of MSC on routine light microscopy (×10, Eclipse, Nickon, Tokyo, Japan). (A)** ‘Control 24 h’ group cells, **(B)** ‘Si 24 h’ group cells, and **(C)** ‘SiB 24 h’ group cells.

When silica-based NPs (Si, SiB) were added to the culture medium for 24 h, no changes in either morphology of mesenchymal stem cells (MSCs) (Figure 2) or their viability were detected. The proportion of different types of cells was reported as follows: AnV + cells (early apoptosis), 7.9% to 8.7%; AnV+/PI + cells (post-apoptotic necrosis), 2.8% to 3.2%; and PI + cells (necrosis), 0.9% to 1.2%.

This finding may be evident of lacking any significant impact exerted by these NPs on processes of apoptosis and necrosis being performed in the cultivated cells.

### Cell stiffness

**Figure 3:**
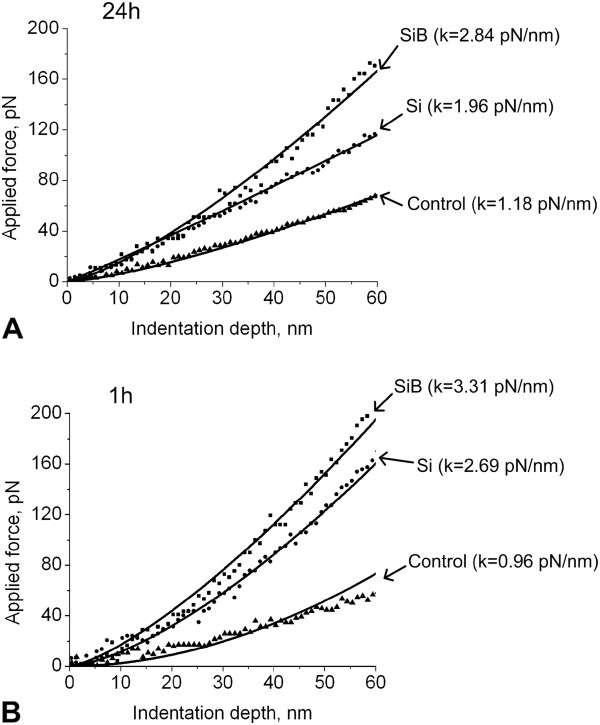
**Typical force curves, obtained during measurements of the cell stiffness (depending on the study group). (A)** Cultivation with nanoparticles lasted 24 h. **(B)** Cultivation lasted 1 h.

The results of the cell stiffness measurements (see Table 1) demonstrated an increase in stiffness by 63% and 136% (as compared to ‘Control 24 h’ group) after being cultured for 24 h in the presence of Si (‘Si 24 h’ group) and SiB (‘SiB 24 h’ group) NPs, respectively (*p* < 0.05) (see Figure 3A).

**Table 1:** Stiffness of cells (pN/nm)

**Study groups/duration of cultivation**		**Control**	**Si**	**SiB**
24 h	*M* ± *D*	1.20 ± 0.11 (*n* = 27)	1.95 ± 0.13* (*n* = 28)	2.83 ± 0.16*^/$^ (*n* = 30)
	*M* ± SE	1.20 ± 0.04 (*n* = 27)	1.95 ± 0.05* (*n* = 28)	2.83 ± 0.05*^/$^ (*n* = 30)
1 h	*M* ± *D*	0.95 ± 0.08* (*n* = 31)	2.7 ± 0.7^@/$^ (*n* = 27)	3.3 ± 1.1^@/#/%^ (*n* = 28)
	*M* ± SE	0.95 ± 0.04* (*n* = 31)	2.66 ± 0.11^@/$^ (*n* = 27)	3.27 ± 0.14^@/#/%^ (*n* = 28)

*n*, number of cells investigated; *M*, mean value; *D*, dispersion; SE, standard error of the mean; *p < 0.05 as compared to Control 24 h group, ^$^*p* < 0.05 as compared to Si 24 h group, ^@^*p* < 0.05 as compared to Control 1 h group, ^#^*p* < 0.05 as compared to Si 1 h group, ^%^*p* < 0.05 as compared to SiB 24 h group.

Moreover, on completion of 1-h cultivation, changes were found to be more pronounced in comparison to the corresponding control (‘Control 1 h’ group); the cell stiffness increased by 181% in the ‘Si 1 h’ group and by 247% in the ‘SiB 1 h’ group (*p* < 0.05) (see Figure 3B).

**Figure 4:**
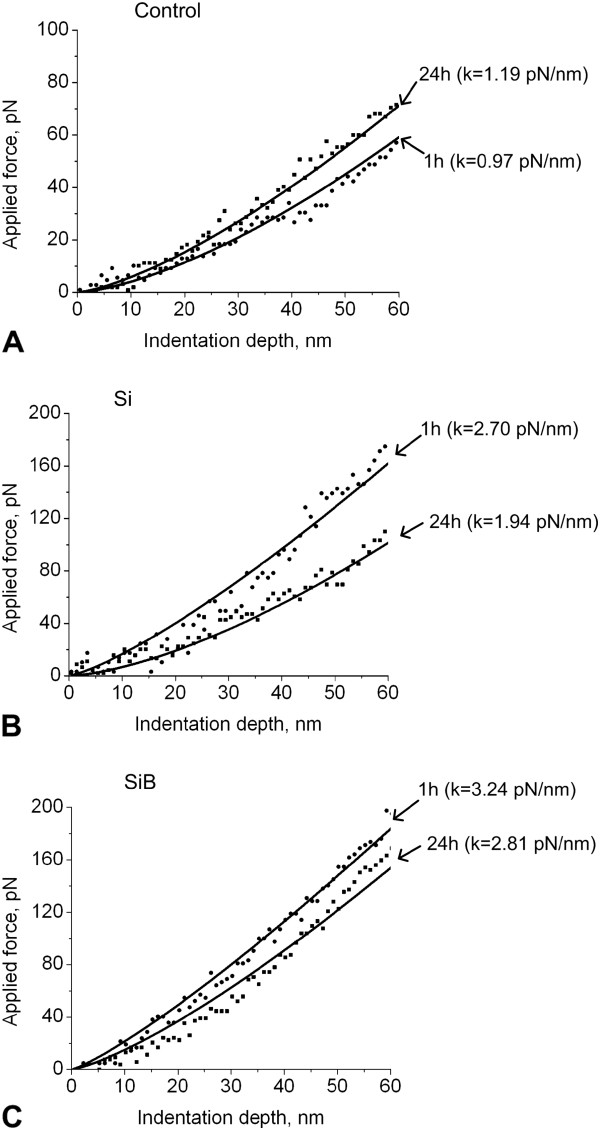
**Typical force curves, obtained during measurements of the cell stiffness (depending on the duration of cultivation). (A)** Cells of the control groups, **(B)** cells cultured with Si nanoparticles, and **(C)** cells cultured with SiB nanoparticles.

It should be mentioned that within 1 h after the medium was changed, the cell stiffness (Control 1 h) was found to be 20% lower (*p* < 0.05) as compared to cells for which the medium was changed within 24 h before measurements (Control 24 h) (see Figure 4A).

At the same time, the stiffness of cells cultured with Si NPs for 1 h (Si 1 h group) was reported to be 36% higher (*p* < 0.05) in comparison to the cells which were cultured in the presence of the same NPs for 24 h (Si 24 h group) (see Figure 4B).

A similar situation was noted when cells were cultured in the presence of SiB NPs; the stiffness of cells cultured with SiB NPs for 1 h (SiB 1 h group) was reported to be 16% higher (*p* < 0.05) in comparison to the cells that were cultured in the presence of the same NPs for 24 h (SiB 24 h group) (see Figure 4C).

Moreover, the dispersion of stiffness values for cells that were cultured in the presence of different types of NPs for 1 h was significantly higher than the dispersion of stiffness values for cells that were cultured in the presence of different types of NPs for 24 h. The dispersion of the cell stiffness values was found to be similar across both control groups.

### F-actin content

**Figure 5:**
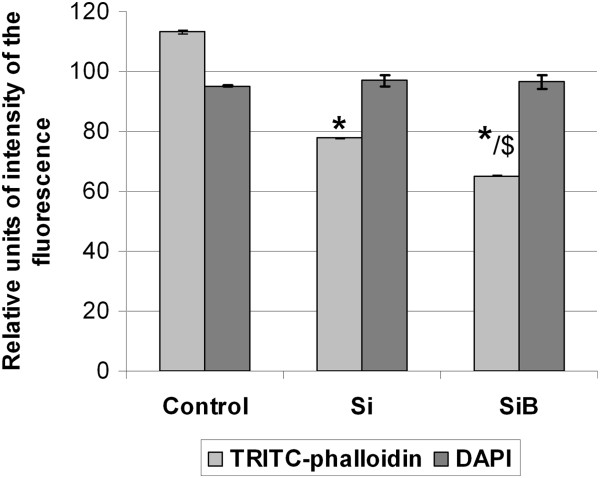
**TRITC-phalloidin and DAPI fluorescence intensity in the following study groups.** Control 24 h is marked with ‘Control’ sign on this image, Si 24 h marked with ‘Si’, and SiB 24 h marked with ‘SiB’. *p < 0.05 in comparison to the Control 24 h group; ^$^*p* < 0.05 as compared to the Si 24 h group.

**Figure 6:**
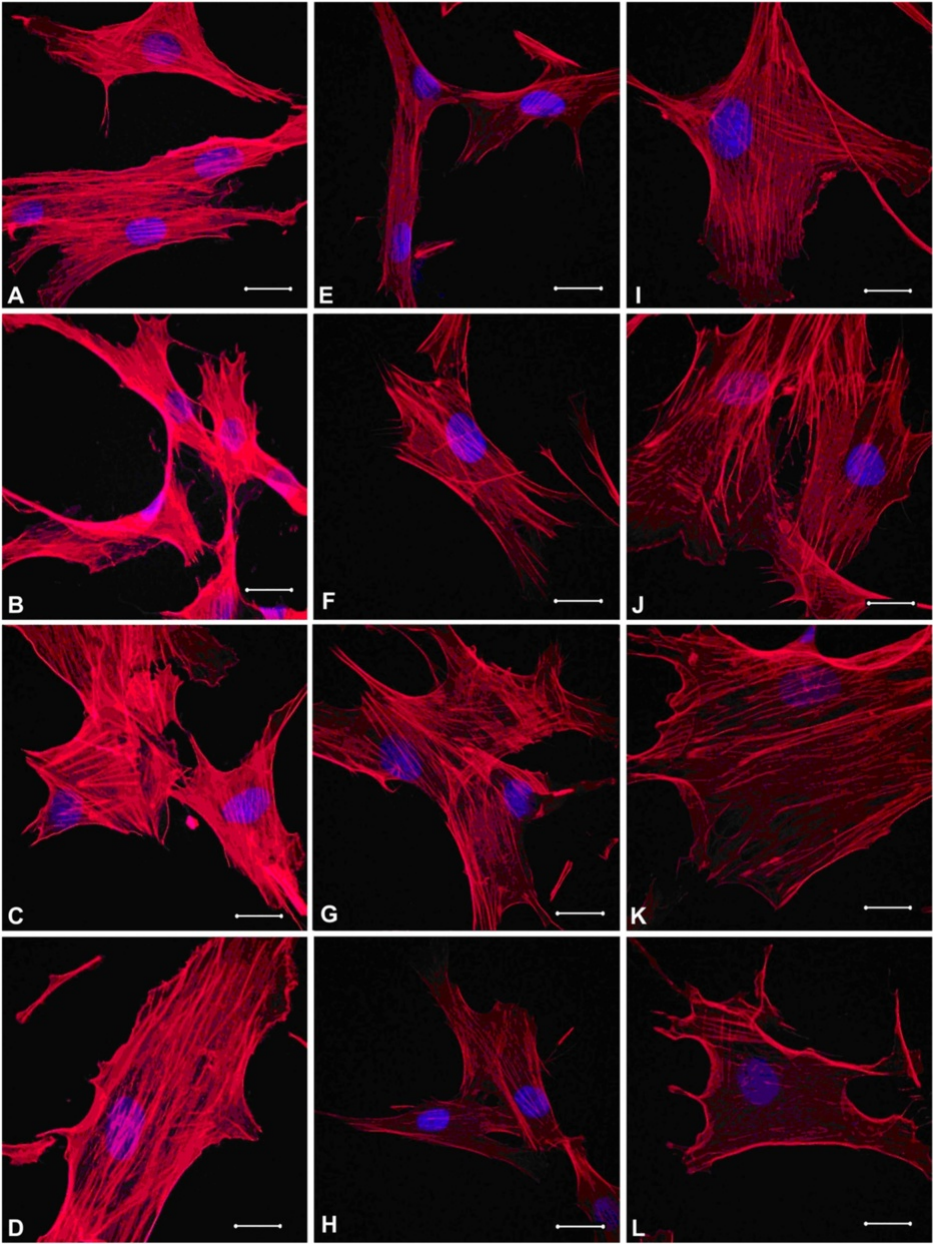
**Typical appearance of MSCs with DNA labeled with blue DAPI staining and F-actin detected with red TRITC-phalloidin staining. (A, B, C, D)** Cells of the ‘Control 24 h’ group: F-actin fibers are mainly longitudinally packed ending with some thickening at the periphery of the cells; diameter of fibers is changeable; in some cases, cell exfoliation from the substrate can be noted; and nuclei are located in the central part of the cells. The length of the marker line is 20 μm. **(E, F, G, H)** Cells of the ‘Si 24 h’ group: some isolated transversally arranged actin filaments appear besides mainly longitudinally packed fibers. The length of the marker line is 20 μm. **(I, J, K, L)** Cells of the ‘SiB 24 h’ group: transversally arranged filaments are detected to a much greater extent within cells, and numerous actin filaments terminate with clavate growing. The length of the marker line is 20 μm.

**Figure 7:**
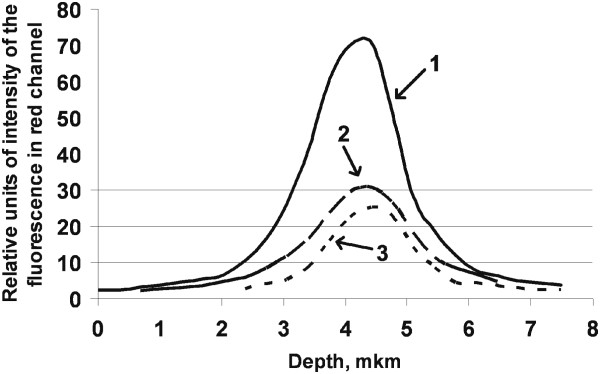
**Distribution of TRITC-phalloidin fluorescence intensity, measured at different depths of the mesenchymal stem cells (z-stacks).** Fluorescence intensities in the control group (curve #1) and after cultivation with Si (curve #2) and SiB nanoparticles (curve #3) normalized according to their maximum values. No peaks of Gaussian distribution shifted. This finding is highly suggestive of even distribution of actin filaments across the depth of a cell in all study groups.

TRITC-phalloidin fluorescence intensity (which normally directly correlates with F-actin content) reduced gradually according to the following order: Control 24 h - Si 24 h - SiB 24 h. The values of this parameter were 31% and 42% lower in the Si 24 h group and SiB 24 group, respectively, as compared to the Control 24 h group (*p* < 0.05) (see Figure 5). Moreover, no changes in DAPI fluorescence intensity were detected in either study group as compared to the control level. It should be noted that some structural reorganization of the actin cytoskeleton was detected upon completion of cultivation with NPs: actin filaments are packed mainly longitudinally within cells of the Control 24 h group (Figure 6A,B,C,D), isolated transversally arranged filaments appeared within cells of the Si 24 h group (Figure 6E,F,G,H), and transversally arranged filaments are detected to a much greater extent within cells of the SiB 24 h group, as compared to the cells of the Si 24 h group (Figure 6I,J,K,L).Evaluation of actin filament distribution across the height of a cell showed that actin fibrils were found to be mainly centrally located in all study groups (Control 24 h, Si 24 h, SiB 24 h) without diffusion towards the surface of a cell (see Figure 7).

## Discussion

It has previously been shown that silica-based nanoparticles do not alter the viability of cultivated lymphocytes on completion of a 24-h exposure. However, the boron-doped NPs were able to cause some changes in mitochondria, lysosomal compartment, and the content of active oxygen forms within cells [19]. We obtained similar results in terms of the cells' viability in our study, in which progenitor cells (mesenchymal stem cells) served as the study object. The amount of cell death that occurred through early and late apoptotic pathways after cultivation with Si and SiB NPs as well as the distribution of the cell death pathways did not differ from the control group.

However, the mechanisms of interaction between cells and NPs have not yet been fully clarified.

Hence, we decided to measure some mechanical characteristics (particularly cell stiffness) of cells cultured in the presence of NPs using AFM. The obtained experimental data indicates that the estimated values of cell stiffness are fully comparable with human non-muscle cells, such as fibroblasts, lymphocytes, mesenchymal stem cells, osteoblasts, and endothelial cells [21,23-25]. At the same time, there is a difference between the mean values of stiffness after 1 and 24 h of incubation. We suggest that this time effect is connected to the specific origin of the NPs, as well as to the concentration effect [6].

When measured at the indentation depth of 60 nm, cell stiffness reflects uppermost the organization of the membrane and cortical cytoskeleton structure. But the data from which the stiffness of the cortical cytoskeleton is determined is very contradictory. For instance, Pelling et al. [26] investigated the impact of nocodazole (the substance that causes tubulin depolymerization) on the mechanical properties of NIH3T3 cells. It was shown that the mechanical properties of cell membranes decreased without any significant differences in values depending on use of different (serum-containing and serum-free) mediums. Costa et al. [23] studied the mechanical properties of human aorta endothelial cells (HAEC). The measurements were conducted in liquid using the contact mode with an indentation depth of 20 nm. The authors found that there were two types of cells, which differed in values of their Young's modulus: one type of cell had the tensile modulus of 5.6 ± 3.5 kPa, while the other had one of 1.5 ± 0.76 kPa. However, after treating with cytochalasin B (at a concentration of 4 μM), no differences in mechanical properties of cells were detected and the values of their Young's modulus (0.89 ± 0.46 kPa) were significantly lower than before processing with this actin-destroying agent. Collinsworth et al. [27] also demonstrated that processing with cytochalasin D resulted in a reduction of cell stiffness, while treatment with colchicine (a microtubule-destroying agent) did not cause any changes in stiffness.

Stiffness changes may result from a number of reasons: localization of the point for measurements, changes in protein content (particularly F-actin/G-actin ratio), changes in structural organization of the cortical cytoskeleton, and modifications of the cell surface.

According to the data obtained by Mathur et al. [21], the values of cell stiffness were significantly higher in the projection of the nucleus, rather than at the periphery of the cells. But the authors used indentation depths of 1 μm in their measurements. As we used the indentation depth of 60 nm in our estimations, all the changes observed in our study are unlikely to be related to localization of the point for measurements.

It can be suggested that reduction of the cell stiffness in the cells of the Control 1 h group (as compared to Control 24 h group) may be related to mechanical load on the cortical cytoskeleton due to the changes in the medium. Such changes resulted in transient alterations of its structure and, as a result, in detection of slight (but statistically significant) reduction of stiffness.

However, what exactly influences the elevation of the cell stiffness when cultured with different NPs and what determines the differences in stiffness values in terms of the types of particles remain unclear.

On the one hand, Cai et al. [24] showed that the mechanical characteristics of cells can serve as a diagnostic parameter (for instance, in the analysis of lymphocyte degeneration). Normal human lymphocytes and human T lymphoblastic Jurkat cells were investigated. Atomic force microscopic images showed that the cell profiles (particularly surface striations) were similar in both types of cells. However, the stiffness of normal lymphocytes is 2.28 ± 0.49 mN/m, and it is 4.32 ± 0.3 mN/m for Jurkat lymphocytes. While studying pathological forms of erythrocytes in patients with hereditary spherocytosis, glucose-6-dehydrogenase deficiency, thalassemia, and anisocytosis, Dulinska et al. [28] demonstrated that their stiffness was found to be increased as compared to normal cells. Lekka et al. [29] assessed the stiffness of erythrocytes in patients with confirmed diagnoses of coronary disease hypertension and diabetes mellitus and compared the values with the corresponding parameters of erythrocytes in healthy volunteers. The authors demonstrated that mean values of the erythrocytes' Young's modulus and the dispersion of its values were statistically higher in patients with diabetes mellitus and in smokers as compared to healthy subjects. Moreover, the Young's modulus of erythrocytes increased with the age of patients. In other words, the detected increments of the cell stiffness resulted from interaction with silica-based NPs, which may serve as one of the earliest markers of their cytotoxic effect.

On the other hand, most of the available data on interactions between NPs and cells suggest that the values of the Young's modulus decrease under such conditions [3]. But it should be mentioned that we measured the cell stiffness in our study, not the Young's modulus. It is connected with the fact that the assessment of the Young's modulus comes to the solution of the Hertz problem [30]. But the solution of the Hertz problem was developed for uniform and isotropic material. Cell structure is not uniform and isotropic. This is why we suggested that Hooke's stiffness is more acceptable for measurements with short indentation depths, such as those used in our study.

We proposed that there are changes in the stiff structure of the cortical cytoskeleton (with F-actin mainly contributing in its formation), so we decided to determine its content using TRITC-phalloidin, for which the intensity of fluorescence within the cell volume was assessed using confocal microscopy.

The obtained data suggested that F-actin content in the submembranous compartment decreased gradually within the following line: ‘Control’ - ‘Si’ - ‘SiB’ , as the intensity of phalloidin fluorescence dropped in the same manner. Nevertheless, the direct fluorescence quenching seems to be unlikely, as no concomitant decrease of DAPI fluorescence intensity was reported in our studies. Furthermore, actin can be transferred from the membranous to the cytoplasmic fraction in the form of F-actin, with further dissociation of the latter to G-actin, as well as directly in the form of G-actin. Transient increase of G-actin content, in turn, may initiate some signaling pathways (for instance, some SRF-dependent pathways) [16]. The results of our study on levels of TRITC-phalloidin fluorescence after cultivation of cells with NPs are in full compliance with available literature data [4].

**Figure 8:**
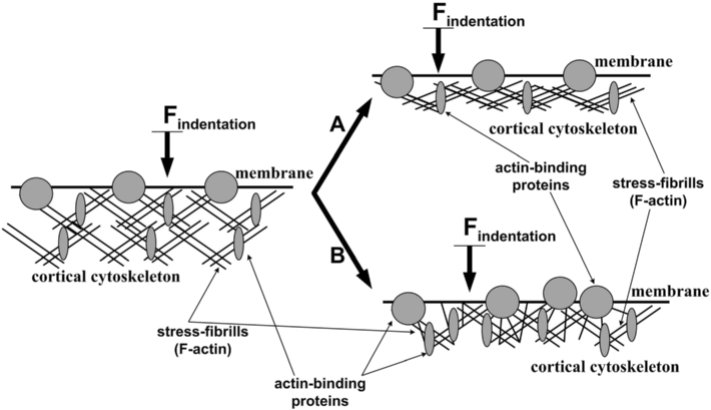
**Possible scheme of cortical cytoskeleton reorganization resulting in stiffness elevation under concomitant decrease of F-actin content. (A)** The quantity of stress fibrils decreases, but they are ‘packed’ more densely within the cell. **(B)** Stress fibrils are within the same distance from each other as initially (before challenge), but the content of actin-binding proteins is found to be increased in the cortical cytoskeleton (probably due to their recruitment within the membrane that resulted from interaction between membrane and nanoparticles); moreover, the transversally oriented actin filaments appearing in the cells may create additional ‘stiffening ribs’. The proposed mechanism is only hypothetical and needs to be checked experimentally.

Therefore, it can be supposed that the detected elevation of stiffness is not related to the increase of the quantity of stress fibrils. Tubulin cytoskeleton, probably, may contribute to stiffness increase [26]. However, its contribution will be very small when using relatively shallow indentations in stiffness measurements [27].We can now offer a hypothesis about how the reorganization of the submembranous cytoskeleton (under conditions of F-actin content decrease) results in cell stiffness increasing. When the number of actin filaments drops, but they are ‘packed’ more densely within the cell, the stiffness may increase (see Figure 8 (A)). In another case, visual increase of the quantity of the transversally oriented actin filaments may result in stiffness increments of a structure (see Figure 8 (B)). The proposed mechanism is only hypothetical and needs to be checked experimentally.

Furthermore, modifications of cell surface may contribute to stiffness increase. It is well known that changes in membranous cholesterol content, resulting in the reorganization of cholesterol rafts, lead to changes in structural organization of the cortical cytoskeleton [31-33]. Increase of dispersion of stiffness values for cells that were cultured for 1 h as compared to dispersion of stiffness values for cells that were cultured for 24 h suggests that interactions between cells and particles are in their active phase. The cell stiffness was higher after 1-h cultivation as compared to their values after 24-h cultivation, potentially due to at least a two-step process: first, the particles bind to the surface of cells, modifying their mechanical properties, and then they diffuse inside the cells, modifying the structure of the cortical cytoskeleton.

However, in analyzing the reasons for changes in cell stiffness, it should be noted that glass was used as the substrate for cell cultivation and, further, for stiffness measurements, which, in accordance with the literature data [34-36], may result in uncharacteristic reorganizations of the cytoskeleton, decreasing the measured cell stiffness. At the same time, all groups of cells were cultivated under the same conditions; thus, we can discuss with confidence about the observed changes in mechanical properties of cells on completion of their cultivation with NPs.

## Conclusions

In this study, we demonstrated that cultivation of mesenchymal stem cells in the presence of Si and SiB NPs results in elevation of their cortical cytoskeleton stiffness with concomitant decrease of F-actin content. The mechanism for such elevation is still unclear, but, probably, it can be related to structural reorganization (for example, increase of the number of ‘cross-links’ between stress fibrils). However, mechanisms inducing such changes are underinvestigated and, probably, may be linked to modifications of the cell surface and/or interactions with the membrane.

## Competing interests

The authors declare that they have no competing interests.

## Authors' contributions

IVO carried out the atomic force microscopy, participated in the design of the study, and drafted the manuscript. SVB carried out the confocal microscopy and helped to draft the manuscript. ANS participated in the design of the study and carried out the cell cultivation and the estimation of the cells' viability. LBB conceived the study and participated in its design and coordination and helped to draft the manuscript. All authors read and approved the final manuscript.
